# Akirin Is Required for Muscle Function and Acts Through the TGF-β Sma/Mab Signaling Pathway in *Caenorhabditis elegans* Development

**DOI:** 10.1534/g3.119.400377

**Published:** 2019-11-25

**Authors:** Richard Bowman, Nathan Balukoff, Amy Clemons, Emily Koury, Talitha Ford, Kunal Baxi, Carlos Egydio de Carvalho, Sarit Smolikove

**Affiliations:** * Department of Biology University of Iowa, Iowa City, IA 52240 and; † Department of Biology, University of Saskatchewan, 112 Science Place, Saskatoon, SK, S7N 5E2

**Keywords:** *C. elegans*, Akirin, AKIR-1, TGF-β, Sma/Mab

## Abstract

Akirin, a conserved metazoan protein, functions in muscle development in flies and mice. However, this was only tested in the rodent and fly model systems. Akirin was shown to act with chromatin remodeling complexes in transcription and was established as a downstream target of the NFκB pathway. Here we show a role for *Caenorhabditis elegans* Akirin/AKIR-1 in the muscle and body length regulation through a different pathway. Akirin localizes to somatic tissues throughout the body of *C. elegans*, including muscle nuclei. In agreement with its role in other model systems, Akirin loss of function mutants exhibit defects in muscle development in the embryo, as well as defects in movement and maintenance of muscle integrity in the *C. elegans* adult. We also have determined that Akirin acts downstream of the TGF-β Sma/Mab signaling pathway in controlling body size. Moreover, we found that the loss of Akirin resulted in an increase in autophagy markers, similar to mutants in the TGF-β Sma/Mab signaling pathway. In contrast to what is known in rodent and fly models, *C. elegans* Akirin does not act with the SWI/SNF chromatin-remodeling complex, and is instead involved with the NuRD chromatin remodeling complex in both movement and regulation of body size. Our studies define a novel developmental role (body size) and a new pathway (TGF-β Sma/Mab) for Akirin function, and confirmed its evolutionarily conserved function in muscle development in a new organism.

Akirin is a nuclear protein that acts in several metazoan pathways regulating development and cell proliferation (Nowak and Baylies 2012). Akirin is present as one copy in invertebrates and has duplicated in the vertebrate ancestor resulting in two homologs (Akirin1 and Akirin2) encoding proteins with ∼70% sequence similarity (Macqueen and Johnston 2009). The two vertebrate proteins have mostly overlapping functions, however in mouse Akirin2 is essential for development while Akirin1 is not (Goto *et al.* 2007). Conditional knockout of Akirin2 in mouse determined it is involved in development of limb, brain, and muscle tissues (Bosch *et al.* 2016; 2018; 2019). The first published role for Akirin described its function in the immune system. Depletion of Akirin in *Drosophila* leads to dysfunction of the innate immune system resulting in sensitivity to bacterial infection (Goto *et al.* 2007). In mouse, loss of Akirin2 also leads to increased sensitivity to bacterial infection due to defects in B-cell differentiation and reduced cytokine production (Goto *et al.* 2007; Tartey *et al.* 2015). Recent work has pointed to a role of *C. elegans* Akirin in innate immune response (Polanowska *et al.* 2018). Akirin also has a conserved function in muscle differentiation in both fly and rodent model systems. Akirin depletion in flies leads to defects in muscle development (Nowak *et al.* 2012). Both Akirin proteins are important for muscle function in vertebrates. Overexpression of mouse Akirin1 (also named Mighty) promoted myocyte differentiation and increased chemotaxis in myoblast cell line, and overexpression of Akirin2 (also named FBI) enhanced the proliferation of porcine and mouse muscle cell lines (Marshall *et al.* 2008; Salerno *et al.* 2009; Chen *et al.* 2017; Bosch *et al.* 2019). Knockdown of Akirin1 or Akirin2 decreased the proliferation in porcine and mouse muscle cells and conditional knockout of Akirin2 perturbed muscle development *in vivo* in the mouse (Chen *et al.* 2017; Bosch *et al.* 2019). Akirin2, but not Akirin1, was also studied in the context of tumor promotion. Inhibition of Akirin2 was associated with the loss of tumorigenic potential as evidenced by increased anchorage-dependent growth, reduced metastasis and tumor size (Komiya *et al.* 2008; 2014). Akirin also has been shown to play a role in meiosis in *C. elegans*, but a germline function has not yet been reported for Akirin in any other organism (Clemons *et al.* 2013).

The diverse roles Akirin plays are consistent with its molecular function as a member of several protein complexes regulating transcription. *Drosophila* Akirin and mouse Akirin2 act in the NFκB pathway to regulate expression of genes required for the innate immune response (Goto *et al.* 2007; Bonnay *et al.* 2014; Tartey *et al.* 2014; 2015). In this pathway, Akirin2 was shown to interact with BAF60 which is part of the SWI/SNF chromatin remodeling complex (Tartey *et al.* 2014). Akirin’s role in chromatin remodeling was also shown in *Drosophila*, where Akirin forms a complex with BAP55-BAP60-Brahma to regulate the immune response (Bonnay *et al.* 2014) and with Twist-BAP60-Brahma during myogenesis (Nowak *et al.* 2012). The SWI/SNF complex (BAF53a-BRG1) also interacts with Akirin to regulate neural development in *Xenopus* (Liu *et al.* 2017). The SWI/SNF chromatin remodeling complex is composed of two distinct sub complexes; BAP and PBAP. Studies of the innate immune response and neurodevelopment implicate Akirin as part of the BAP complex, while in myogenesis, Akirin acts in both the BAP and the PBAP complexes. Interestingly, there is no direct evidence that *C. elegans* Akirin interacts with SWI/SNF. Instead, Akirin was shown to both physically and genetically interact with the NuRD chromatin remodeling complex (Polanowska *et al.* 2018). NuRD is an ATP-dependent chromatin remodeling complex that contains a histone deacetylase (Torchy *et al.* 2015). In *C. elegans* NuRD is implicated in many biological processes including vulva development, somatic differentiation, asymmetric cell division, meiotic recombination, and regulation of lifespan (Unhavaithaya *et al.* 2002).

While the developmental roles of Akirin are via chromatin remodeling, its cell proliferation functions point to a different protein complex involving Akirin2 (FBI). In these studies Akirin2 physically interacted with 14-3-3β to promote repression of transcription (Komiya *et al.* 2008). Although Akirin’s role in transcription is conserved, its mode of action is not; it can either lead to the activation (Nowak *et al.* 2012; Bonnay *et al.* 2014) or the repression (Komiya *et al.* 2008; Akiyama *et al.* 2013) of transcription of different target genes. The lack of any known domains in Akirin, including DNA binding or transcription activation domains, suggests that Akirin is an adaptor protein that contributes to the assembly of complexes required for transcriptional regulation.

The Transforming Growth Factor-β (TGF-β) pathways are conserved metazoan intercellular signaling pathways (Zhang *et al.* 2017) that regulate developmental processes leading to the establishment of body plans and cell fates (Gumienny and Savage-Dunn 2013). In *C. elegans* there are two TGF-β pathways which play a number of roles during development and in the adult tissue (Savage-Dunn and Padgett 2017). In the TGF-β Dauer pathway, signaling occurs through DAF-7 ligand, eventually affecting downstream targets (DAF-5 and DAF-3) to induce the metabolic changes associated with the dauer stage (Morita *et al.* 2001). The TGF-β Sma/Mab pathway positively regulates body size through the activity of the *sma* (Small) genes, as well as male morphology (Male ab normal) through the Sma/Mab pathway via the ligand DBL-1/CET-1 (Savage *et al.* 1996; Morita *et al.* 1999). The downstream components of TGF-β Sma/Mab signaling pathway are not completely known, with *sma-9* as the known transcription factor involved (Savage-Dunn *et al.* 2003; Gumienny and Savage-Dunn 2013). The TGF-β Sma/Mab pathway is involved in the downregulation of autophagy genes (Guo *et al.* 2014), the maintenance of lipid droplets size (Clark *et al.* 2018) and the immune response [regulate antimicrobial peptide production (Zugasti and Ewbank 2009)]. TGF-β signaling was shown to require SWI/SNF-dependent chromatin remodeling in some organisms (Nowak and Baylies 2012), but the connection between TGF-β signaling and chromatin remodeling in *C. elegans* is unknown.

In *C. elegans*, no developmental or mitotic function has been described for Akirin/AKIR-1 aside from its function in the innate immune response (Polanowska *et al.* 2018). *C. elegans* Akirin/AKIR-1 plays an important role in the disassembly of the synaptonemal complex in meiosis [a meiotic protein complex essential for chromosome segregation and the establishment of sister-chromatid cohesion (Clemons *et al.* 2013; Bowman *et al.* 2019)]. Here we investigate the role of Akirin during development and in the maintenance of somatic tissues. We find that AKIR-1 plays a role in muscle function, consistent with its previously described activities in other organisms. *akir-1* mutants show defects in the localization of muscle lineage markers in the embryo and in the adult as well as deterioration of muscle function that affects movement. Depletion of *akir-1* also leads to reduced body size. Finally, we show that *akir-1* functions via the TGF-β signaling Sma/Mab pathway, consistent with the elevated markers of autophagy found in *akir-1* mutants.

## Materials and Methods

### Strains

*C. elegans* strains in the N2 background were maintained in standard growth conditions at 20° on NGM plates.

Alleles used in this study: *akir-1*(*gk528*) I, *swsn-2.2*(*tm3395*) I, *swsn-7*(*tm4263*) II, *sma-2*(*e502*) III, *sma-3*(*e491*)III, *mep-1*(*ok421*) IV, *lin-40*(*ok906*) V, *dbl-1*(*wk70*) V, *sma-1*(*ru18*) V, *sma-9*(*wk55*) X, *sma-9*(*iow109*) X, *lon-2*(*e678*) X. Reporters, tagged lines and arrays used: *akir-1*(*iow37*[FLAG::akir-1]) I, *jjIs2437*[pCXT51(RAD-SMAD) + LiuFD61(mec-7p::mRFP)] II, *oxIs322* [myo-2p::mCherry::H2B + myo-3p::mCherry::H2B + Cbr-unc-119(+)II, *ayIs6* [*hlh-8*::GFP fusion + *dpy-20*(+)]X, *ctIs40* [*dbl-1*(+) + sur-5::GFP] X, iowEx3[*akir-1*; *rol-6*], *vkEx1093* [nhx-2p::mCherry::lgg-1]. Balancers used: *hT2* [*bli-4*(*e937*) let-?(*q782*) *qIs48*] (I;III), *mIn1** [**mIs14* *dpy-10**(**e128**)] II*, *nT1** [**qIs51**] (IV;V)*.

### Somatic rescue of akir-1 via extra chromosomal array

*iowEx3[**akir-1*; *rol-6**]*, was produced by injecting *akir-1**(**gk528**)/**hT2* worms with 30ng/μl of a 2.7 kb *akir-1* rescue fragment amplified from N2 genomic DNA with primers flanking the gene, including its promoter (from the ends of the CDS of the flanking genes E01A2.5 and E01A2.1). pRF4 (*rol-6**(**su1006**))* was used as co-injection marker (50 ng/μl).

### CRISPR/Cas9 genome modification

CRISPR/Cas9 Genome modification was performed as in Bowman *et al.* 2019 with the modifications indicated here. The crRNA used was targeting the following site: GTATCGTTGAGACGAAGAAC. The *wk55* allele contains a pre-mature stop mutation in *sma-9*. To introduce an allele equivalent to *sma-9**(**wk55**)* an ssODN was used as repair template. This ssODN contained a single nucleotide change of C to T in position 4899 and silent mutations that remove the PAM site (sequence: TGAATTTGTCGTTTATGGGAATGGTATCGTTGAGAtGAAGAACGGATCAACAGAAATTTTTCAAATACACAACGGCCA). The point mutation was confirmed by PCR and sequencing using the primers: Forward GCAGTACAAGATGGAACTTTATCG, Reverse GGAGAGGTGGAGATTGAGG.

### RNA interference (RNAi)

All plasmids used for RNAi were in the pL4440 vector transformed to HT115 strain. The RNAi clone for *swsn-4* was obtained from the RNAi library. 1-1.3kb cDNA for *swsn-1*, *swsn-3*, and *swsn-6* were amplified from cDNA library and cloned into pL4440 Gateway vector using Gateway technology with the following primers: *swsn-1*: Forward GGGGACAACTTTGTACAAAAAAGTTGGCatgaagaagggagcgtcg Reverse GGGGACAACTTTGTACAAAAAAGTTGGgccacttttcgtaagcc. *swsn-3*: Forward GGGGACAACTTTGTACAAAAAAGTTGGCatgtcatctttccgtcatcc Reverse GGGGACAACTTTGTACAAAAAAGTTGGttattcttccattttttcttccg. *swsn-6*: Forward GGGGACAACTTTGTACAAAAAAGTTGGCatgagtggagcagtttatggag Reverse GGGGACAACTTTGTACAAAAAAGTTGGttacgggcacttcttctc.).

Worms were age matched by bleaching gravid wild type and *akir-1**(**gk528**)* animals and placing the recovered eggs onto unspread agar plates and incubated overnight at 20°. Worms at the L1 stage were washed from the hatching plate with sterile M9 and placed in 10μl drops onto RNAi plates containing the indicated clones, and pL4440 and *bli-1* were used as controls, then allowed to grow to day 1 adults (2 days) in the dark at 20° at which time they reached adulthood and their body length was measured.

### Thrashing assay

The number of thrashes per minute per worm was obtained by recording worm movement in drops of M9 buffer. The video was played at reduced speed to facilitate counting. Each individual thrash was counted as a full sinusoidal movement of the worm from beginning point in one direction and returning to the original position, then doubled to account for the full number of thrashes as in Peden *et al.* 2013. Worm thrashing video was acquired using a dissecting microscope and an 8mp camera. The worms were suspended in 35μl M9 for 1 min prior to recording, and were recorded for 1 min. The total number of worms counted for each genotype at each age can be found in the figure legend.

### Muscle integrity assay

Hermaphrodites at day 5 post-L4 were frozen with liquid nitrogen and dried in a SpeedVac. After lyophilization the worms were re-suspended in 20µl Rhodamine-Phalloidin extracted from methanol and diluted in S-mix. The worms were incubated in the dark and washed with 5% BSA/1xPBST and suspended in 1xPBS. The worms in suspension were placed on an agar bed and mounted with Vectashield containing DAPI.

Myocyte 2D images from day 3 and day 5 post-L4 worms were acquired at 20X at myocyte juncture sites at varying points along the length of each worm. The gap distance between myocytes was measured by counting the number of pixels between actin filaments at various points along individual myocytes from the tip of one to the tip of another and averaged to obtain the distance between individual myocytes. The total number of myocytes measured for each genotype at each age can be found in the figure legend.

### Egg retention assay

Hermaphrodites in L4 stage were selected and collected on a single agar plate with a lawn of OP50 bacteria and incubated overnight at 20°. The resulting day 1 and day 2 post-L4 hermaphrodites were fixed using Carnoy’s fixation and mounted with Vectashield with DAPI. Number of embryos in the uterus was counted for each worm.

### Vulva burst assay

To determine vulva burst of each genotype, single L4 worms were placed on seeded NGM plates and scored visually for bursting vulvas for 3 days. Worms were transferred every day to avoid confusion with their progeny.

### Embryonic lethality assay

To determine embryonic lethality of each genotype single L4 worms were placed on seeded NGM plates and allowed to lay eggs for a 15 hr period. The worms were moved to a fresh NGM plate and this was repeated for a three-day period. Number of eggs and L1 larva were counted for each genotype examined and the embryonic lethality was calculated as: ((#eggs-#L1)/#eggs)*100.

### Embryo staining

A plate of gravid adults was bleached and eggs were deposited on a positively charged slide and covered with a coverslip (1.5 hr post bleaching for bean and comma stage, 6.5 hr for twofold and 7.5 hr for threefold embryos). The embryos were freeze-cracked on dry ice and slides placed in cold 100% methanol at -20° before being transferred to cold acetone at -20°. Slides were then successively incubated in acetone 70%, acetone 50%, and acetone 30% at 4°, rinsed in 1xPBS and washed in fresh 1xPBS at room temperature. After blocking in 0.5% BSA, slides were incubated overnight with primary antibody and for 2 hr with secondary antibody, both at room temperature. Slides were washed between incubations in PBST and mounted in Vectashield with DAPI. All antibodies used were from the Developmental Studies Hybridoma Bank.

### Body length measurement

For analysis of body length in adults, worms were picked as L4s based on shape of the vulva and 24 hr later worms were analyzed. For analysis of body length in L3, worms were synchronized by bleaching and fixed following 50 hr incubation at 20°. All worms were adults based on the shape of the vulva at the time of analysis. Full body length 2D images were acquired with a 20X lens. To convert pixels to distance we used a cell counting chamber, applied the following formulae, and determined that 1 pixel was equal to 1μm.

$$P_{x}\left[\frac{\mu m}{px}\right]=\frac{Real\;distance\;in\;x\;direction[\mu m]}{Distance\;in\;pixels=\Delta x=x_{2}-x_{1}[px]};\;P_{y}\left[\frac{\mu m}{py}\right]=\frac{Real\;distance\;in\;y\;direction[\mu m]}{Distance\;in\;pixels=\Delta y=y_{2}-y_{1}[py]}$$

The body length of each separate worm was measured from head to tail following the center of the body. The total number of animals measured for each genotype can be found in the figure legend.

### LGG-1 foci counts

Intestinal cell 2D images from live day 1 post-L4 worms were acquired with a 100X lens. The images were taken at the approximate center of the intestinal cells of immobilized worms. The number of LGG-1 foci present in both intestinal cells presented in an image were counted. The total number of worms imaged for each genotype at each age can be found in the figure legend. Images are of ∼1$mm^{2}$.

### BODIPY counts

One day old adults were washed in PBS and resuspended in 0.5ml water (final volume). 0.5ml of 8% paraformaldehyde (PFA) (4% final) was added and worms were incubated 15 min at room temperature. Worms were frozen in liquid nitrogen and immediately thawed. Thawing-freezing was repeated twice followed by one wash in M9 buffer. 500µl of 1µg/ml BODIPY 493/503 was added and worms incubated for 1 hr at room temperature. Following incubation, worms were washed 3 times in M9 buffer, mounted on slides and analyzed.

### Nile red counts

∼800 1-day old adults were resuspended in 1 ml of water to which 50 µl of freshly prepared 10% paraformaldehyde was added. The worms were immediately frozen in liquid nitrogen and thawed. After two freeze/thaw cycles, worms were allowed to settle, and paraformaldehyde solution was removed. One ml of 1 µg/ml Nile Red in M9 buffer was added to the pellet and worms incubated for 15–30 min at room temperature, with occasional gentle agitation. Worms were allowed to settle, washed once with M9 buffer, and allowed to settle again. Fixed worms were mounted onto 2% agarose pads for microscopic observation and image acquisition.

### Whole worm fixation

Hermaphrodites in L4 stage were collected on a single agar plate with a lawn of OP50 bacteria and incubated overnight at 20°. The resulting day 1 post-L4 hermaphrodites were fixed using Carnoy’s fixation and mounted with Vectashield with DAPI.

### Live worm imaging

Live imaging was used for detecting mCherry::LGG-1 foci and vulva structure. Hermaphrodites in L4 were collected on a single agar plate with a lawn of OP50 bacteria and incubated overnight at 20°. Day 1 post-L4 hermaphrodites were placed in M9 buffer on a slide prepared with a 4% agarose pad. The worms were immobilized with beads diluted with OP50 bacteria in liquid LB media or with sodium azide.

### RAD-SMAD reporter assay

This assay was performed as in Tian *et al.* 2013. Worms on OP50 plates were allowed to grow until all food was gone and the adults were gravid. Each plate was washed with M9 buffer, the buffer/worm solution was placed into a 1.5ml tube, and pelleted at 2000 rpm for 1 min. The supernatant was removed and the worms were washed in M9 buffer twice more to remove residual bacteria. After the final wash, 1ml of bleach/NaOH solution was added. After 10 min with periodic vortexing, worms were pelleted at 3000 rpm for 1 min and the eggs washed 3 times with 1ml M9 buffer. After the final wash, the eggs collected on plates without food and incubated at 15° for 24 hr. Hatched L1 larvae were transferred to NGM-OP50 plates and incubated at 20° for 48 hr (L3 stage) when they were live imaged on a Delta Vision deconvolution microscope at 100X magnification. From each image, GFP fluorescence intensities in hypodermal cells were measured in 5 nuclei and normalized using background measurements collected outside of the nucleus, in the cytoplasm of the tissue (FIJI software).

### Male tail imaging

Wild type and *akir-1**(**gk528**)* males were imaged at 100x on DIC while immobilized with beads and an agar pad. Images were taken with an Andor Zyla sCMOS camera on a Leica DMi8 microscope. Males were obtained from populations of mating isogenic strains. *C. elegans* males are formed by random non disjunction of the X-chromosome (which can be facilitated by 4-hour incubation at 32°). Once a male (X0) was isolated it was crossed to hermaphrodites (XX) of the same genotype. 50% of crossed progeny were males. Mating plates were maintained for the duration of the experiments from which males were collected.

### Statistics

The data were compiled into GraphPad 8.1 and significance assessed with Fisher’s exact test, ANOVA, or two-tailed Mann-Whitney *U*-test with a 95% CI, as indicated in the text and figure legends. * 0.01 ≤ *P* ≤ 0.05, ** 0.001 ≤ *P* < 0.01, *** 0.0001 ≤ *P* < 0.001, ****P < 0.0001.

### Data availability

Strains are available upon request. The authors state that all data necessary for confirming the conclusions presented in the article are represented fully within the article. Supplemental material available at figshare: https://doi.org/10.25387/g3.10795307.

## Results

### AKIR-1 is a nuclear protein expressed in numerous somatic cells

**Figure 1:**
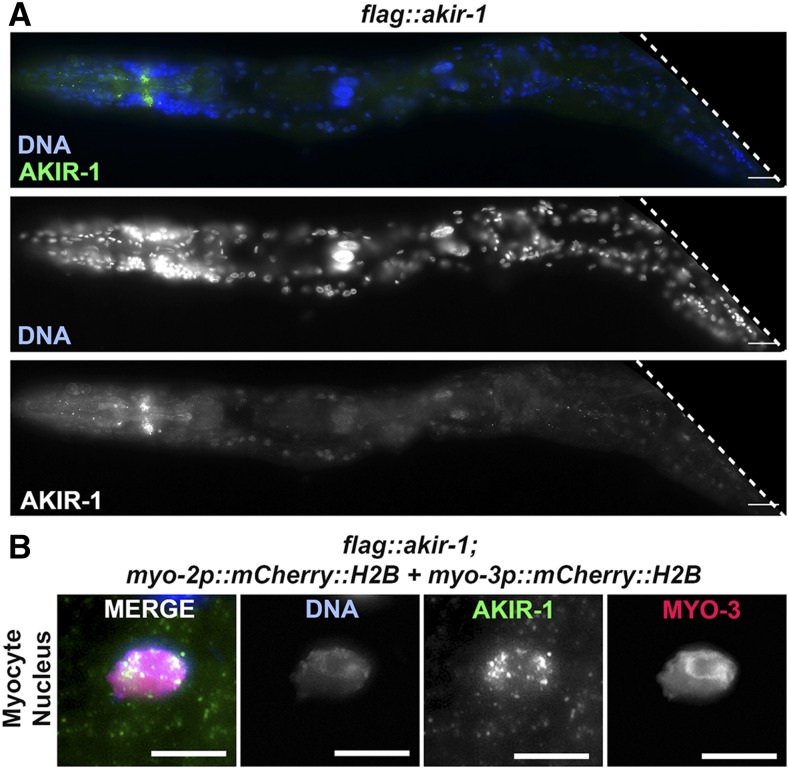
AKIR-1 is localized to somatic nuclei, including that of muscle cells. A) Whole body staining of wild type *C. elegans* for DAPI (blue) and AKIR-1 (green, anti-FLAG) (top) and DAPI and AKIR-1 channel separately (middle and bottom respectively). B) Myocyte nucleus with mCherry::MYO-3 (red) stained for DAPI (blue) and AKIR-1 (green, anti-FLAG), and single channels from left to right showing localization patterns of DAPI, AKIR-1, and MYO-3. Scale bars 2μm.

*C. elegans* encodes a single Akirin protein, named AKIR-1. AKIR-1 was previously shown to be required for the proper disassembly of the synaptonemal complex during meiosis and for the innate immune response (Clemons *et al.* 2013; Polanowska *et al.* 2018). Due to its conserved developmental function in fly and vertebrate model systems we hypothesized that Akirin also plays a role in development and is expressed in somatic tissues of *C. elegans*. Transcriptional reporter gene assays indicated that the Akirin promoter is turned on in many somatic tissues [wormbase.org, (Polanowska *et al.* 2018)] but the expression pattern of the AKIR-1 GFP-tagged protein is different and found only in two tissues: the L3 epidermal nuclei and the diakinesis nuclei of the adult germline (Polanowska *et al.* 2018). It is possible that this discrepancy is due to weak expression of AKIR-1 in somatic tissues under the detectable GFP fluorescence threshold. If so, signal amplification using antibodies could potentially bypass this problem. We performed immunofluorescence using an anti-FLAG antibody in whole worms using a strain with a FLAG-tagged N-terminal insertion in the native *akir-1* locus (Bowman *et al.* 2019). This tag was shown to have an improved germline staining compared to two GFP -tagged lines (Hefel and Smolikove 2019). Consistent with the reported localization of Akirin in other species, FLAG::AKIR-1 was detected in somatic nuclei in many tissues (Figure 1A), including in muscle cells where it colocalized with a muscle nuclear reporter [*mCherry*::*H2B* driven by *myo-2* and *myo-3* promoters, (Zacharias *et al.* 2015) Figure 1B].

### AKIR-1 is required for muscle function

**Figure 2:**
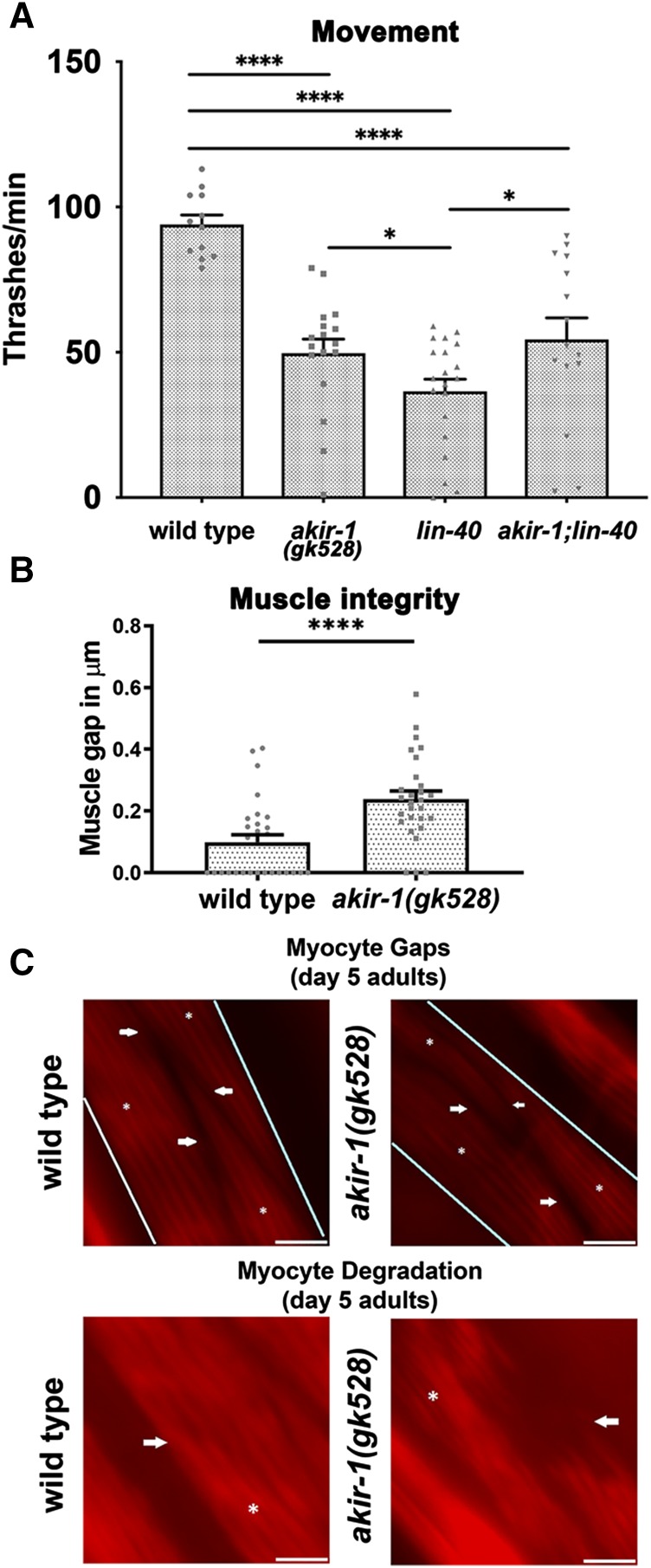
AKIR-1 is required for muscle function. A) Quantification of movement was done by counting full sinusoidal thrashes of *C. elegans* while suspended in M9. The data plot individual measurements and indicate the mean plus SEM, and n= 12, 17, 20 and 15 for wild type, *akir-1*, *lin-40** and **akir-1**;**lin-40* respectively. B) Muscle integrity was determined by measuring the distance between individual myocytes in the body wall muscle. Individual measurements are plotted with n = 28 and the mean plus SEM is shown. C) Images of body wall muscle myocytes at 5 days post larval adult. For myocyte gap distance, the body wall muscle is indicated within blue lines, individual myocytes are indicated by an asterisk (*), and the myocyte gap is shown by the white arrows. For muscle integrity, individual myocytes are indicated by an asterisk (*) and degraded muscle tissue is indicated with white arrows. Scale bars 10μm. * 0.01 ≤ *P* ≤ 0.05, *** 0.0001 ≤ *P* < 0.001, ****P < 0.0001.

Studies of Akirin in mouse and fly implicated Akirin in muscle differentiation. Consistent with a role in muscle function, *akir-1**(**gk528**)* null mutants show defects in movement, as measured by the rate of body movements (thrashes) in a swimming assay [wild type 94 ± 3, *akir-1* 50 ± 5 thrashes/min (mean± SEM), *P* < 0.0001, Mann Whitney *U*-test (MW), Figure 2A]. Akirin interacts with the SWI/SNF chromatin remodeling complex in fly and mouse (Nowak *et al.* 2012; Bonnay *et al.* 2014; Tartey *et al.* 2014), but in *C. elegans* it was shown instead to physically interact with the NuRD complex (Polanowska *et al.* 2018). This study found four SWSN subunits as potential interactors with AKIR-1, but this was determined to not be statistically significant. Null mutants in the *C. elegans* SWI/SNF core subunits are embryonic and larval lethal, which precludes the analysis of the core complex in muscle development in the adult (Large and Mathies 2014). However, we found that the *swsn-7**(**tm4263**)* null allele (of a member of the PBAF/PBAP complex) produces a few viable homozygous progeny that can be analyzed as adults (Large and Mathies 2014). Defects in movement were not observed in *swsn-7* escapers, suggesting that worm Akirin does not act in muscle movement via the PBAF/PBAP complex (Supplemental Material, Figure S1A). *lin-40* is a member of both NuRD complexes found in *C. elegans*. We measured movement in *lin-40**(**ok906**)* and found that movement is reduced in these mutants to even lower levels than what is found in *akir-1**(**gk528**)* [*lin-40**(**ok906**)* 37 ± 4 thrashes/min, *P* < 0.0001, Mann Whitney *U*-test (MW) Figure 2A]. Interestingly, movement impairment in *akir-1*; *lin-40* double mutants was similar to *akir-1**(**gk528*) (*akir-1**;**lin-40* 54 ± 7), indicating that *akir-1* is epistatic to *lin-40* and that both genes act in the same pathway. These results suggest that *akir-1* acts to support movement through the NuRD complex, and not via the SWI/SNF complex, consistent with studies of the immune system in *C. elegans* (Polanowska *et al.* 2018).

*akir-1**(**gk528**)* null mutants also show defects in the maintenance of muscle tissue integrity. In addition to movement defects observed in young *akir-1* mutants, a widening gap between myocytes is detectable in older (day 5) *akir-1* mutants relative to the wild type (from 0.1 ± 0.02 in WT to 0.24± 0.03μm in *akirin* mutants, *P* < 0.0001, MW, Figure 2B), suggesting premature muscle deterioration. Other more severe defects in muscle integrity further support a role for Akirin in preventing age-dependent sarcopenia (Figure 2C).

**Figure 3:**
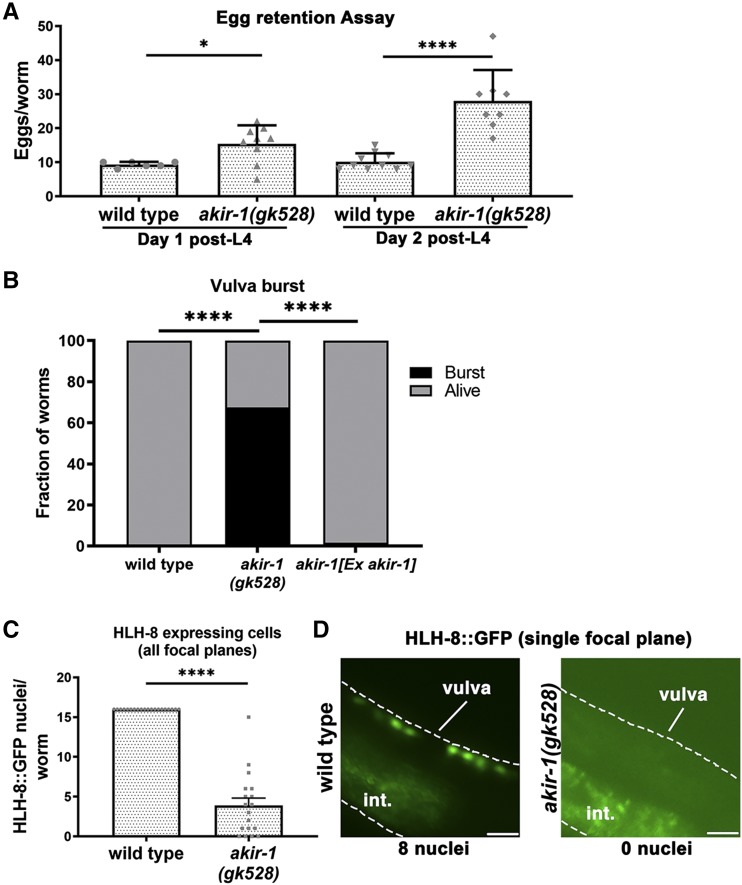
AKIR-1 is required for proper vulvar development and function. A) Eggs retained in the uterus at 1 and 2 days post-L4, mean plus SEM shown. Wild type day 1 n = 6 and day 2 n = 10, *akir-1**(**gk528**)* day 1 n = 9 and day 2 n = 8. B) Rates of vulvar burst were measured. Wild type did not display incidences of vulvar tearing (0%, n = 84), *akir-1**(**gk528**)* counts had n = 77. Expression of AKIR-1 from a wild type *akir-1* gene in an extra-chromosomal array showed rescue of vulvar tearing (2%, n = 57). C) HLH-8 expressing myoblasts present in wild type L4 animals n = 20 and *akir-1**(**gk528**)* n = 18. D) Images of HLH-8 expressing myoblasts in L4 animals in normal, edge-on configuration in wild type and their absence in *akir-1**(**gk528**)*. One focal plane is presented which contains half (8) of the HLH-8 nuclei. Dashed lines are where the borders of the worm body are. The normal position of the vulva is indicated, as is the intestine displaying normal autofluorescence. Scale bars 10μm. * 0.01 ≤ *P* ≤ 0.05, *** 0.0001 ≤ *P* < 0.001.

*C. elegans* typically lays ∼250 eggs over the course of ∼3 days following the switch from the L4 larval stage to the adult stage. Egg laying requires the proper development of the vulva and the function of vulvar smooth muscle cells controlled by the egg-laying neuronal circuitry (White *et al.* 1986). Our previous studies indicated that *akir-1**(**gk528**)* mutants lay fewer (∼10%) eggs as compared to wild type worms (Clemons *et al.* 2013). This reduction in the number of eggs laid could be explained by an impairment in gonad development and/or a defect in egg laying. In principle, defects in egg laying *per se* should not impact ovulation rates and instead should lead to increased retention of eggs in the uterus. Wild type worms at the second day of adulthood retain 10.2 ± 0.8 eggs in the uterus; however, in *akir-1**(**gk528**)* mutants the number of retained eggs increased almost threefold (28 ± 3.2 eggs at day 2, *P* < 0.0001, MW, Figure 3A). Vulvar dysfunction also leads to vulva bursts (tear of the vulva leading to spillage of internal organs). 68% of *akir-1**(**gk528**)* mutants examined had their vulva burst during the first 3 days of adulthood, while this was not seen in wild type worms (*P* < 0.0001 Fisher’s exact test, Figure 3B). Vulva bursts were suppressed in *akir-1* mutants by expressing a wild type copy of *akir-1* from an extra-chromosomal array (from 68 to 2%), indicating that the phenotype was likely caused by the deletion of *akir-1* in the soma (*P* < 0.0001 Fisher’s exact test, Figure 3B).

Lastly, we verified that the vulva defects observed in *akir-1* mutants are likely due to disruption of vulva muscle development. The vulva in *akir-1* mutants formed, though further investigation revealed a developmental defect (Figure S2A). The HLH-8/Twist transcription factor marks the M lineage of sex muscle precursors that give rise in adults to vulva muscles, uterine muscles and body wall muscles (Philogene *et al.* 2012). HLH-8 is normally expressed in the 16 nuclei positioned at the mid body of the L4 stage worm (8 nuclei on each side). In *akir-1**(**gk528**)* mutants, however, the number of HLH-8::GFP positive nuclei was reduced (3.9 ± 0.9, *P* < 0.0001, MW, Figure 3C, D). Taken together, our data point to a role of AKIR-1 in muscle development and function in adult *C. elegans*, consistent with its role in other organisms.

### AKIR-1 is required for proper embryonic development of muscle tissue

**Figure 4:**
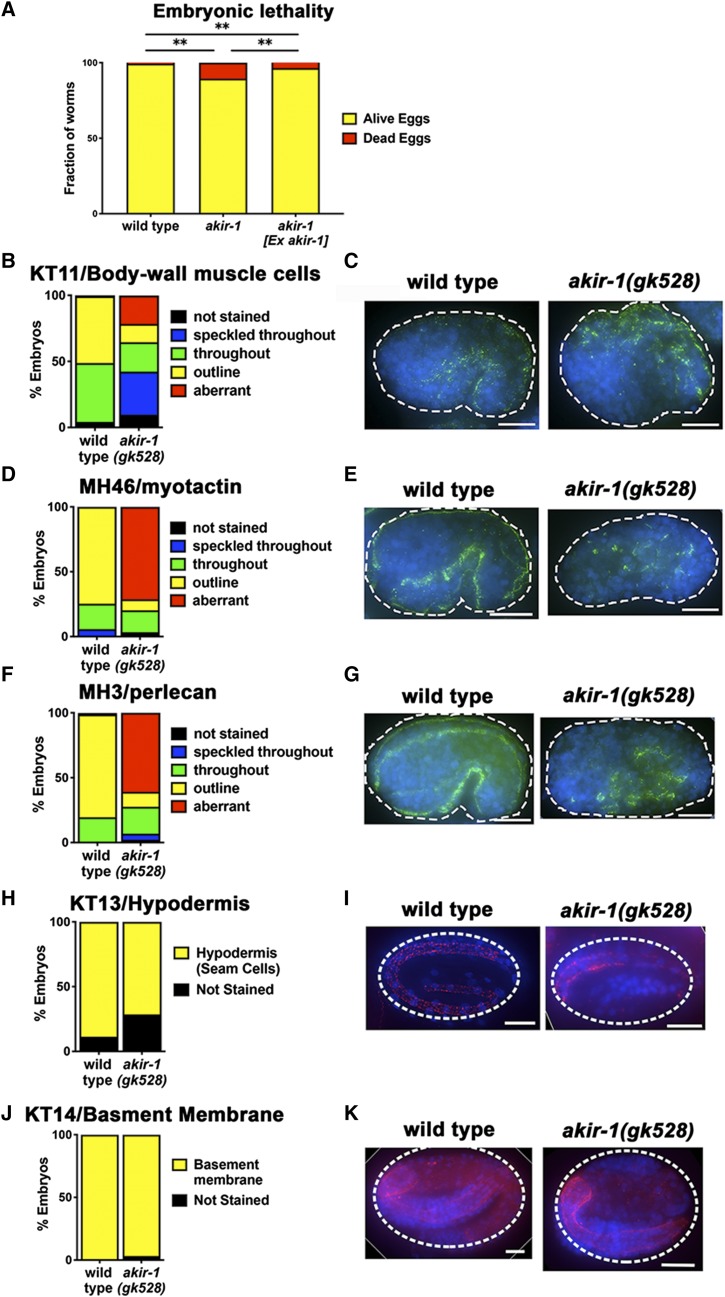
AKIR-1 is required for proper embryonic development. A) Embryonic lethality (eggs laid not developed to adult worms). Wild type n = 2298, *akir-1**(**gk528**)* n = 598. *akir-1**(**gk528**)* with wild type *akir-1* in an extra-chromosomal array had an embryonic lethality of 3.6% (n = 2800, *P* < 0.0001, Fisher’s Exact test). B-G) Immunolocalization of antibodies for embryonic tissues: B-G embryos at the bean stage, H embryos at the 1 to 3 fold stage, and J embryos and the 2 to 3 fold stage. B) Percent of embryos with the indicated staining pattern for KT11. Wild type n = 168, *akir-1**(**gk528**)* n = 144. C) Antibody staining of KT11; representative images of the most frequent staining pattern observed for each genotype. D) Percent of embryos with the indicated staining pattern for MH46. Wild type n = 138, *akir-1**(**gk528**)* n = 59. E) Antibody staining of MH46; representative images of the most frequent staining pattern observed for each genotype. F) Percent of embryos with the indicated staining pattern for MH3. Wild type n = 184, *akir-1**(**gk528**)* n = 87. G) Antibody staining of MH3; representative images of the most frequent staining pattern observed for each genotype. H) Percent of embryos with the indicated staining pattern for KT13. Wild type n = 44, *akir-1**(**gk528**)* n = 7. I) Antibody staining of KT13; representative images of the most frequent staining pattern observed for each genotype. H) Percent of embryos with the indicated staining pattern for KT14. Wild type n = 51, *akir-1**(**gk528**)* n = 30. I) Antibody staining of KT14; representative images of the most frequent staining pattern observed for each genotype. Scale bars 10μm. Statistics: A ** 0.001 ≤ *P* < 0.01., B, D and F outlined staining *vs.* all other Fisher’s exact test, all significant *P* < 0.001, H and J stained *vs.* not Fisher’s exact test, both non-significant *P* = 0.2422, *P* = 0.3704 respectively.

We have previously shown that *akir-1**(**gk528**)* mutants have a small increase in embryonic lethality that may be attributed at least in part to defects in the meiotic divisions (Clemons *et al.* 2013). However, embryonic lethality may also be the result of a post-meiotic perturbation of AKIR-1 functions in somatic blastomeres. Extrachromosomal arrays are normally silenced in the germline but readily expressed in somatic tissues. Consistent with a contribution of somatic AKIR-1 activity in embryogenesis, expressing *akir-1* from an extrachromosomal array partially suppressed the embryonic lethality of *akir-1**(**gk528**)* mutants (from 10.5 to 3.6%, *P* < 0.0001, wild type control showed a 0.6% lethality, Fisher’s Exact test, Figure 4A). SYP-1 is an essential component of the synaptonemal complex and in its absence chromosomes missegregate. *syp-1* embryo lethality is therefore fully explained by a defect in meiotic chromosome non-disjunction. Careful examination of the time of embryonic arrest indicated that a significant proportion of *akir-1* embryos progress further developmentally as compared to *syp-1* embryos, indicating that most *akir-1* embryos die from defects unrelated to chromosome nondisjunction (Table 1). In addition, and reminiscent of Akirin depletion in meiosis, mitotic divisions in *akir-1**(**gk528**)* embryos are also delayed (Table 2). Together, these results support a dual role for AKIR-1 in regulating embryonic viability by supporting gametogenesis as well as embryonic development.

**Table 1:** Embryonic Arrest

	Embryonic stage arrest						
	Pre-comma	Not full comma	Comma	Older than comma	Pinched (asymmetrical)	hatched w/defect	n
*syp-1*	5.2%	48.3%	19%	3.4%	24.1%	0%	**58**
*akir-1*	3.8%	49%	20.8%	13.2%	0%	13.2%	**53**

Percent of embryonic arrest at the indicated developmental stages (right to left). The numbers indicate the percent of embryos that arrested at each of the indicated stages for *syp-1* and *akir-1* mutants.

**Table 2:** Mitotic Divisions

	2 cell to bean	Bean to comma	Comma to 1.5	1.5 to 2	n
wild type	17911+/− 682	1999+/− 652	1599+/− 359	1277+/− 285	**8**
*akir-1*	23262+/− 6207	1946+/− 915	2250+/− 589	1861+/− 460	**11**

Average time of mitotic divisions in seconds to the indicated developmental stages (right to left) +/− standard deviation for wild type and the *akir-1* mutant.

To identify the tissues affected by AKIR-1 depletion during development, we analyzed the localization of markers for different cell types/developing tissues in wild type and arrested (likely dead) *akir-1**(**gk528**)* embryos. For this analysis we used antibodies marking specific tissues at the bean embryonic stage (Francis and Waterston 1991; Takeda *et al.* 2008). Markers of muscle lineages were mislocalized in the *akir-1**(**gk528**)* mutant embryos (Figure 4). KT11 is a monoclonal antibody that stains body-wall muscle cells (Takeda *et al.* 2008). In wild type embryos, the KT11 signal can be detected in about half (51%) of the cells in the bean stage embryo, while this tissue-specific localization was found in only 14% of *akir-1**(**gk528**)* bean stage mutant embryos (*P* < 0.0001 Fisher’s exact test, Figure 4B). MH46 recognizes myotactin, a hypodermal protein involved in muscle-cell adhesion. Myotactin distribution on the membrane of hypodermal cells is dependent on correct development of body wall muscle cells (Francis and Waterston 1991). In wild type embryos at the bean stage, the MH46 signal is observed in 75% of the cells, while only 8% of *akir-1**(**gk528**)* mutants displayed this localization pattern (*P* < 0.0001 Fisher’s exact test, Figure 4D). Finally, MH3 recognizes perlecan, a cell-surface molecule synthesized by muscle cells (Francis and Waterston 1991). 79% of cells in wild type embryos showed localization of MH3 in the bean stage, while only 11% of *akir-1**(**gk528**)* bean stage mutant embryos exhibited this localization pattern (*P* < 0.0001 Fisher’s exact test, Figure 4F). We observed no significant differences in the localization of markers for hypodermis or basement membrane (KT13 and KT14, Figure 4H-K). Altogether these results indicate that although an *akir-1* deletion leads to a partly penetrant phenotype, AKIR-1 does play an important role in embryonic development consistent with its function in muscle maintenance in the adult (Figure 1).

### AKIR-1 is a component of the TGF-β signaling pathway that regulates body size in C. elegans

**Figure 5:**
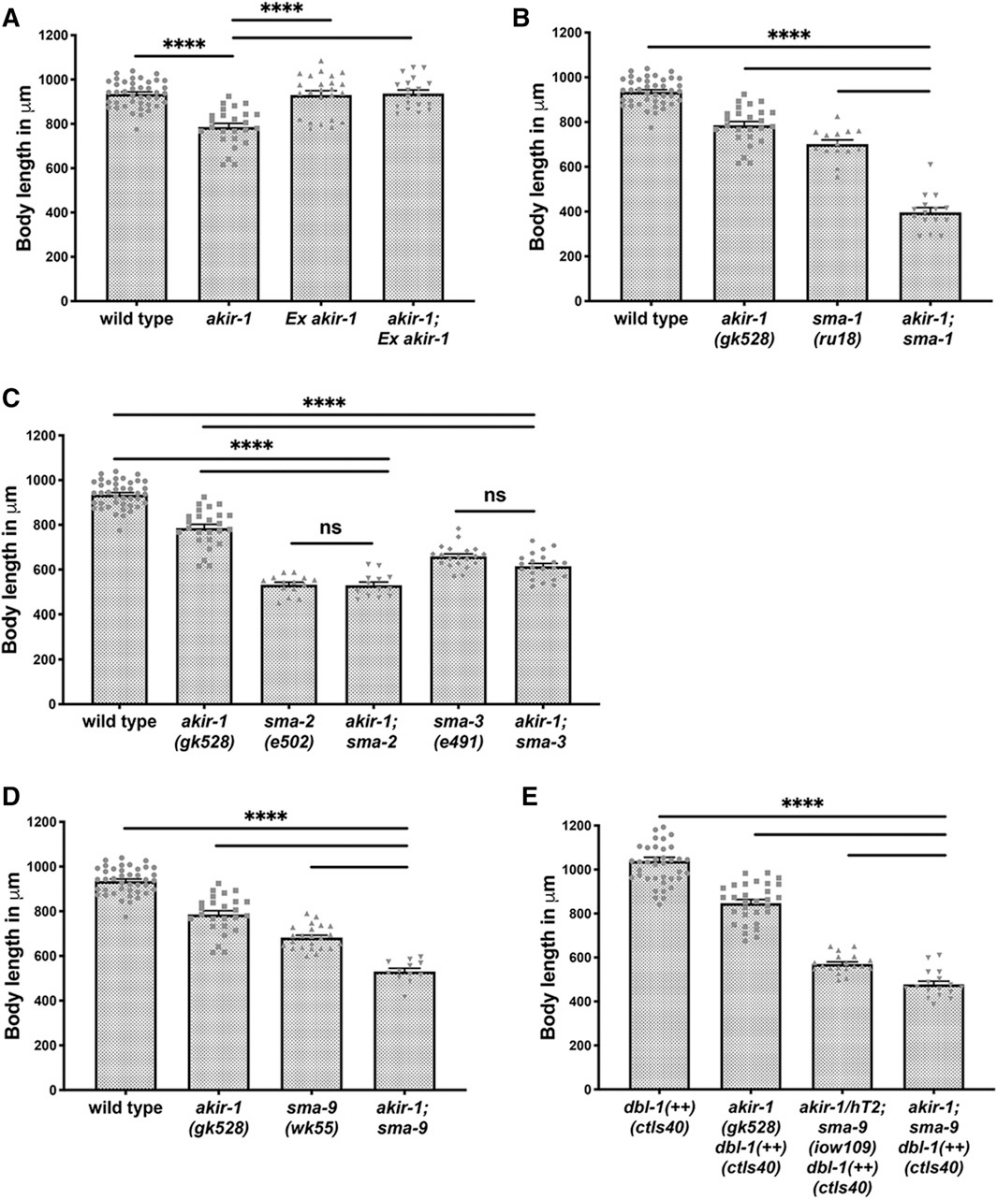
AKIR-1 is a component of the TGFβ pathway and is required for proper *C. elegans* body length. Body length in wild type, *akir-1**(**gk528**)* and TGFβ (Sma/Mab) loss-of-function mutants. Quantification of body lengths, individual measurements plotted with mean plus SEM shown. A) Wild type n = 41, *akir-1**(**gk528**)* n = 25. Overexpression of AKIR-1 via the presence of a wild type *akir-1* gene in an extra-chromosomal array results in no significant change in body length (mean 930.6μm, SEM 18.98, n = 24), and the wild type *akir-1* gene in the extra-chromosomal array rescues the body length defect when present in *akir-1**(**gk528**)* animals (n = 19). B) *sma-1**(**ru18**)* n = 14, is significantly shorter than wild type (*P* < 0.0001, MW *U*-test) but not *akir-1**(**gk528**)*. (*P* = 0.0786, MW *U*-test). *akir-1**(**gk528**);**sma-1**(**ru18**)* n = 15. C) *sma-2**(**e502**)* n = 14, *akir-1**(**gk528**);**sma-2**(**e502**)* n = 13. *sma-3**(**e491**)* n = 20, *akir-1**(**gk528**);**sma-3**(**e491**)* n = 21. D) *sma-9**(**wk55**)* n = 23, *akir-1**(**gk528**);**sma-9**(**wk55**)* n = 12, E) *dbl-1**(++)* n = 41, *akir-1**(**gk528**)*; *dbl-1**(++)* n = 30, *dbl-1**(++)*; *akir-1**/hT1*; *sma-9**(**iow109**)* n = 19, *dbl-1**(++)*; *akir-1*; *sma-9**(**iow109**)* n = 17.*** 0.0001 ≤ *P* < 0.001. For simplicity only statistical analysis compared to double mutant is presented in B-E.

Akirin is a downstream component of the NFκB pathway in both mouse and fly systems (Goto *et al.* 2007; Bonnay *et al.* 2014; Tartey *et al.* 2014; 2015). However, *C. elegans* lacks a homolog for NFκB (Irazoqui *et al.* 2010), raising the question of which pathway Akirin is involved with in worms. To explore potential candidate pathways, we examined genetic interactions of Akirin with genes associated with the regulation of body size. *akir-1**(**gk528**)* mutants exhibit shorter body size compared to wild type (935 ± 9 *vs.* 786 ± 17 μm length, *P* < 0.0001, MW, Figure 5A). Similar to *akir-1**(**gk528**)* embryonic lethality, the reduced body size in these mutants can be suppressed by expressing AKIR-1 from an extrachromosomal array (937 ± 16 μm length, *P* < 0.0001, MW, Figure 5A). Only a few pathways have been implicated in determining body size in *C. elegans* (Mörck and Pilon 2006), allowing us to directly examine which of these pathways genetically interact with *akir-1* by using epistasis analysis.

*sma-1* encodes for βH spectrin that controls epithelial cell sheet morphogenesis and is required for proper embryonic elongation (McKeown *et al.* 1998). Predictably, *sma-1* mutants have a significant reduction in body size (702 ± 19 μm). Depletion of AKIR-1 function in *akir-1*; *sma-1* double mutants further reduces body size as compared with *sma-1* single mutants (396 ± 21 μm length, *P* < 0.0001, MW, Figure 5B) suggesting that AKIR-1 and SMA-1 are acting in parallel pathways regulating body length. Next, we examined the relationship between AKIR-1 and the TGF-β Sma/Mab pathway. SMA-2 (Smad1) and SMA-3 (Smad5) are R-Smad homologs that are part of the complex transmitting the signal from the TGF-β receptor to the nucleus (Savage *et al.* 1996). Mutants of both genes show reduced body size (532 ± 11 and 659 ± 11 μm length, Figure 5C). Unlike the additive effect in body length observed in *akir-1*; *sma-1* mutants, the introduction of *akir-1**(**gk528**)* into backgrounds depleted of SMA-2 or SMA-3 function did not reduce body size significantly when compared to any of the single mutants (*akir-1*; *sma-2* 523 ± 14 and *akir-1*; *sma-3* 615 ± 13 μm length, *P* > 0.01, MW, Figure 5C). These results suggest that *akir-1* may be involved in TGF-β signaling.

If AKIR-1 was part of the TGF-β Sma/Mab pathway, considering its nuclear localization, it is likely a downstream component, acting at the transcriptional level in parallel with other TGF-β Sma/Mab pathway proteins that are part of transcription factor complexes. SMA-9 is one of four known transcription factors acting in the TGF-β Sma/Mab pathway [in addition to SMA-2, SMA-3 and SMA-4 (Savage-Dunn *et al.* 2003; Liang *et al.* 2003)]. Accordingly, the body size of *akir-1**(**gk528**)* mutants is further decreased when combined with a *sma-9* mutation (*sma-9* 682 ± 11 and *akir-1*; *sma-9* 530 ± 14 μm length, *P* < 0.0001, MW, Figure 5D), indicating that AKIR-1 interacts with a different TGF-β transcription factor. Since *sma-9* was speculated to act in early development (Liang *et al.* 2003) we performed the same analysis at the L3 stage and obtained similar results (Figure S1C). Over activation of the TGF-β Sma/Mab pathway by overexpression of the DBL-1/BMP ligand leads to an increase in body length (Suzuki *et al.* 1999). We hypothesized that the DBL-1 signal is transmitted to more than one effector protein at the transcriptional level, one of which is AKIR-1. Under this assumption, we expected the reduced body length phenotype of *akir-1**(**gk528**)* mutants to be partially suppressed by DBL-dependent over activation of the TGF-β Sma/Mab pathway (*ctIs40* transgene, *dbl-1* overexpression). As expected, *akir-1*; *dbl-1**(++)* double mutants have longer body size compared to *akir-1**(**gk528**)* single mutants, but are shorter compared to *dbl-1**(++)* mutants [*dbl-1**(++)* 1072 ± 19 and *akir-1*; *dbl-1**(++)* 847 ± 16 μm length, *P* = 0.0167, MW, Figure 5D]. If SMA-9 and AKIR-1 are independent targets of DBL-1, *dbl-1**(++)* will not be able to suppress body size in *akir-1*; *sma-9* double mutants. Indeed, triple mutants containing DBL-1 overexpression *dbl-1**(++)* and both *sma-9* and *akir-1* loss of function alleles, where shorter than DBL-1 overexpression strains that lack either *sma-9* or *akir-1* (Figure 5E).

If AKIR-1 interacts with the SWI/SNF chromatin remodeling complex, as its orthologs do, then SWI/SNF pathway should also be required for body length. Mutants of the core subunits of the SWI/SNF chromatin remodeling complex are completely embryonic lethal, which prevents the analysis of their possible role on body size. RNAi for *swsn* genes (*swsn-1*, *swsn-3* and *swsn-4*) could not be used for this analysis since it did not lead to body length reduction (Figure S1D). *swsn-2.2* encodes a protein with sequence similarity to mammalian BAF60, an accessory subunit of the SWI/SNF chromatin remodeling complex (Large and Mathies 2014). *swsn-2.2**(**tm3395**)* mutants showed no effect on body size (Figure S1B), despite exhibiting high embryonic lethality (Large and Mathies 2014). The lack of a body length phenotype in these escapers may be due to the partial requirement of SWSN-2.2 subunit in this complex. We therefore analyzed *swsn-7**(**tm4263**)*, a null allele of a gene encoding for a member of the PBAF/PBAP complex which shows almost complete embryonic lethality. *swsn-7**(**tm4263**)* escapers displayed a significant reduction in body size (Figure S1B) comparable to that found in *akir-1**(**gk528**)* worms. However, the genetic interaction between the two mutants was additive, indicating Akirin does not act in body size determination via the SWI/SNF PBAF/PBAP complex. Together these results are consistent with AKIR-1 being a downstream component in the TGF-β Sma/Mab pathway and acting independently of SWI/SNF chromatin remodeling complex in determining body size in *C. elegans*.

### akir-1(gk528) mutants show defects associated with perturbation of the TGF-β Sma/Mab pathway

**Figure 6:**
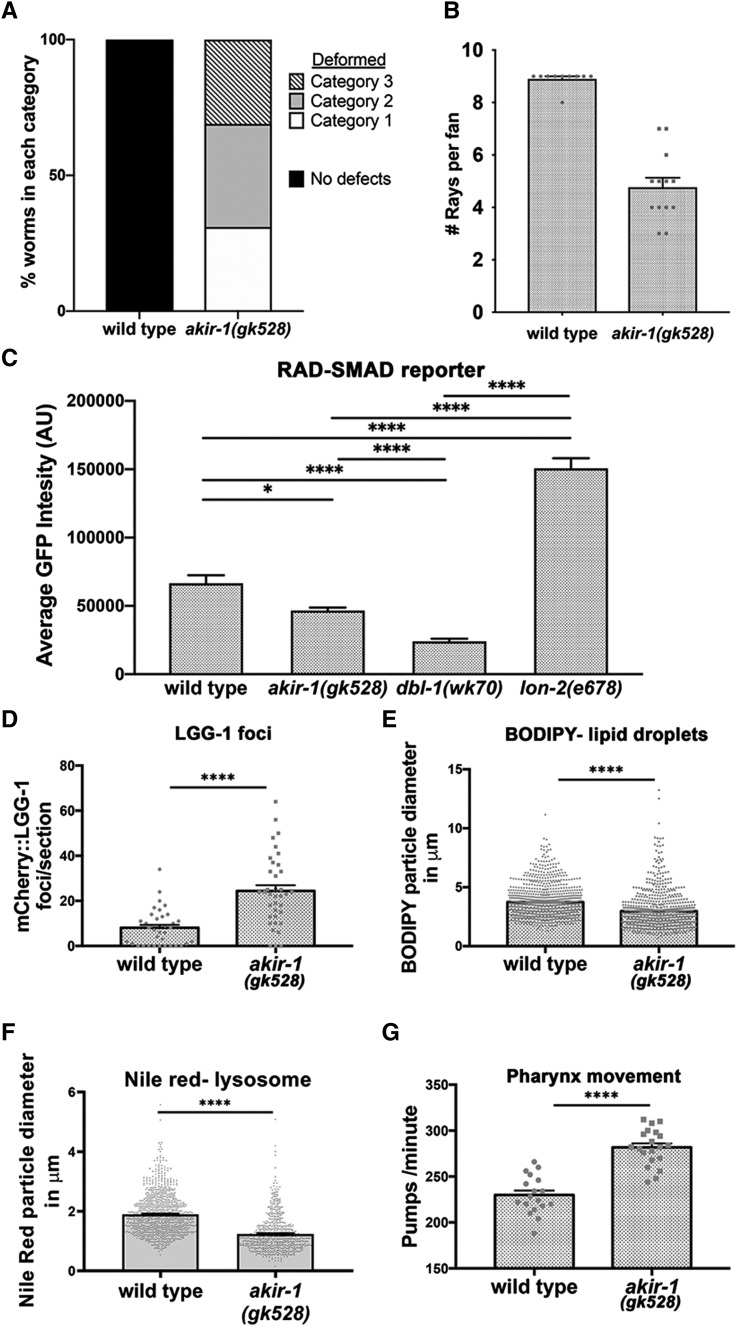
*akir-1* mutants show defects associated with perturbation of the TGF-β pathway. A-B) *akir-1**(**gk528**)* show defects in tail formation. The severity of *akir-1**(**gk528**)* male tail defects were established via the evaluation of number of rays and amount of “fin” present. Severe was classified as less than 2 rays and no fin tissue. Moderate is between 2 and 7 rays with partial presence of fin tissue. Mild was determined by more than 7 rays and substantial fin tissue, but with non-wild type morphology. For A: wild type n = 10, *akir-1* n = 11, *mep-1* ^13^N, *akir-1**;**mep-1* n = 11, For B: wild type n = 11, *akir-1* n = 10, *lin-40* n = 9, *akir-1**;**lin-40* n = 7. C) *akir-1**(**gk528**)* show reduced activation of RAD-SMAD reporter. D) *lgg-1* foci in first two intestinal cells, n = 37. E and F) BODIPY wild type n = 719, *akir-1**(**gk528**)* n = 882 and nile red wild type n = 669, *akir-1**(**gk528**)* n = 882. G) pharyngeal pumping, D-G) Individual measurements plotted with mean plus SEM shown. wild type n = 18, *akir-1**(**gk528**)* n = 20. * 0.01 < *P* ≤ 0.05, *** 0.0001 < *P* ≤ 0.001, ****P < 0.0001.

The TGF-β Sma/Mab pathway is also required for tail development in *C. elegans* males. Mutants in the TGF-β Sma/Mab pathway show defects in male tail morphology, including a reduction in the number of rays, likely due to fusion of rays. We examined tail morphology in *akir-1**(**gk528**)* mutant males and found that most worms exhibited a deformed tail structure with only half of the normal number of rays, consistent with severe defects in tail development (Figure 6A and B and S2B). In our analysis we cannot conclude if AKIR-1 mutants have a phenotype involving ray fusion defects (as seen in TGF-β Sma/Mab mutants) or have morphogenesis defects of the male tail.

The level of activation of TGF-β target genes can be monitored by assaying the fluorescence intensity in a RAD-SMAD responsive reporter strain (Tian *et al.* 2010; Wang *et al.* 2017). Depletion of LON-2, a negative regulator of TGF-β signaling, shows increased expression of the RAD-SMAD reporter, while *dbl-1* mutants in which TGF-β signaling is reduced show decreased expression of the RAD-SMAD reporter (Figure 6C). We examined GFP signal intensity in *akir-1**(**gk528**)* mutants containing the RAD-SMAD reporter and found decreased levels of RAD-SMAD reporter expression. This indicates that AKIR-1 is required for normal activation of TGF-β Sma/Mab pathway target genes. However, the reduction was not as great as observed for *dbl-1* mutants, suggesting that AKIR-1 is likely only one of the factors required for transcriptional activation of genes in this pathway.

TGF-β Sma/Mab pathway mutants have increased expression of autophagy genes (Guo *et al.* 2014) and reduced lipid droplet size. Thus, if AKIR-1 was required for TGF-β signaling, *akir-1**(**gk528**)* mutants should exhibit increased autophagy and reduced lipid droplet and lysosome size. LGG-1/LC3 lipidation is required for the degradation of organelles during autophagy and mCherry::LGG-1 is used as a marker for autophagy levels (Meléndez 2009; Gosai *et al.* 2010). As predicted, *akir-1**(**gk528**)* mutants show increased numbers of LGG-1 foci in the intestine compared to wild type, indicating an increase in autophagy (24 ± 2.5 *vs.* 8.1 ± 1.3 foci/image, respectively, *P* < 0.0001, MW, Figure 6D). BODIPY and Nile Red dyes are used for measurement of lipid droplets and lysosome size, respectively (Brooks *et al.* 2009; Klapper *et al.* 2011). *akir-1**(**gk528**)* mutants show a decrease in BODIPY and Nile Red dyes focus size (*P* < 0.0001 for both, MW, Figure 6E and F), supporting a role for Akirin in lipid metabolism. AKIR-1’s function is therefore consistent with a role in the upregulation of lipid droplets and the downregulation of autophagy through TGF-β Sma/Mab signaling.

Starvation due to reduced pharynx pumping also leads to increased autophagy (Mörck and Pilon 2006). We therefore tested whether *akir-1**(**gk528**)* mutants display feeding defects that could explain the phenotypes above. We find no evidence of decreased pharyngeal pumping in *akir-1**(**gk528**)* mutants. Instead, pumping rates in *akir-1**(**gk528**)* mutants show a small, but significant, increase compared to wild type (282 ± 4 *vs.* 230 ± 5 pumps/minute, respectively, *P* < 0.0001, MW, Figure 6G). Altogether these results are consistent with a model in which AKIR-1 acts as a downstream component of the TGF-β Sma/Mab pathway.

### AKIR-1 regulates body size in C. elegans in the same pathway as LIN-40, a member of the NuRD complex

**Figure 7:**
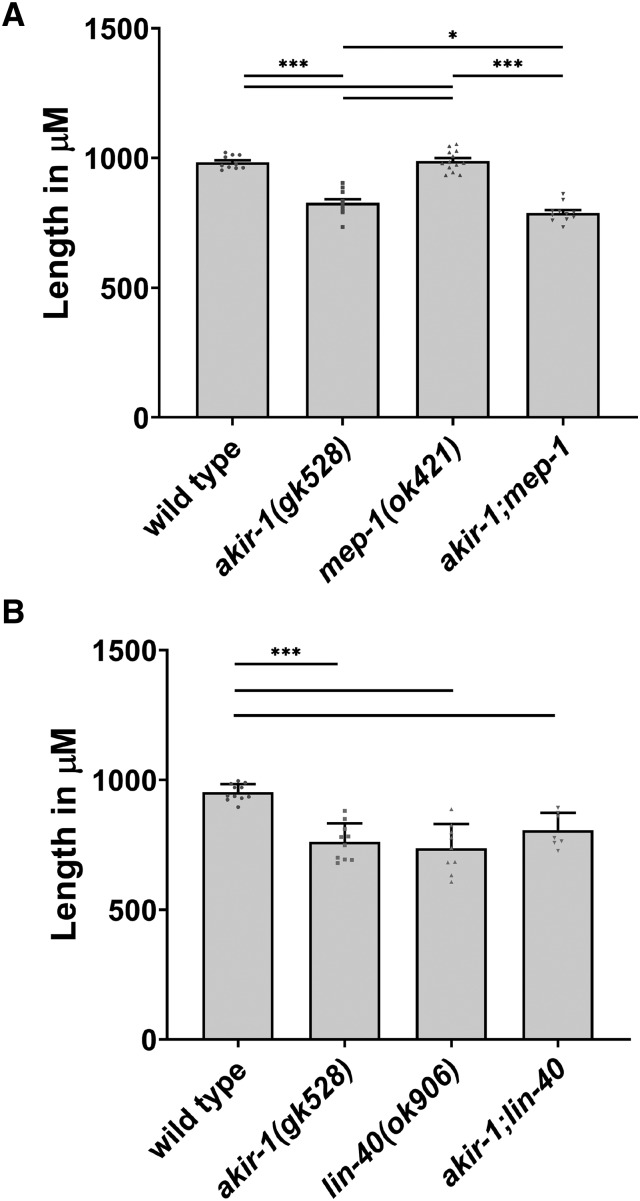
AKIR-1 and NuRD pathway and required for proper *C. elegans* body length. Body length in wild type, *akir-1**(**gk528**)* and NuRD mutants. Quantification of body lengths, individual measurements plotted with mean plus SEM shown. A) wild type n = 11, *akir-1**(**gk528**)* n = 10, *mep-1**(**ok421**)* n = 9, *akir-1**(**gk528**)*; *mep-1**(**ok421**)* n = 7. B) wild type n = 10, *akir-1**(**gk528**)* n = 11, *lin-40**(**ok906**)* n = 13, *akir-1**(**gk528**)*; *lin-40**(**ok906**)* n = 11.* 0.01 < *P* ≤ 0.05, ***0.0001 ≤ *P* < 0.001.

The data above indicate that AKIR-1’s role in muscle function is consistent with it acting in association with the NuRD complex. In addition, we demonstrated that other AKIR-1 somatic functions such as body size determination are involved with TGF-β signaling. To examine whether these two pathways interact we examined body length in two NuRD mutants: *lin-40**(**ok906**)* and *mep-1**(**ok421**)*. *lin-40* is a member of both NuRD complexes (LET-418 and CHD-3 complexes), while MEP-1 only participates in the LET-418 complex (Passannante *et al.* 2010). Interestingly, body size is reduced in *lin-40**(**ok906**)* but not *mep-1**(**ok421**)* mutants, indicating that the NuRD CHD-3 complex but not LET-418 complex is involved in body size regulation (Figure 7). The body size of *akir-1*; *lin-40* double mutants is indistinguishable from the single mutants, indicating that AKIR-1 and LIN-40 likely act in the same pathway. This is consistent with the reported physical interaction between AKIR-1 and NuRD complex members (Polanowska *et al.* 2018). These results indicate that in addition to its immune system role (Polanowska *et al.* 2018), Akirin also functions in development and adult tissue maintenance through the NuRD complex.

## Discussion

*C. elegans* Akirin has been previously shown to act in the germline to mediate proper disassembly of the synaptonemal complex and assembly of the sister chromatid cohesion complex in addition to functioning in the innate immune response (Clemons *et al.* 2013; Polanowska *et al.* 2018; Bowman *et al.* 2019). Here we show that Akirin has other somatic and developmental functions in this model organism. AKIR-1 localizes to the nucleus of somatic cells, and *akir-1* mutants have reduced body size and muscle function. The effect on muscle is apparent in both developmental (embryo, vulva) and post-developmental analysis (muscle integrity and movement). In addition to Akirin’s conserved role in muscle development, we have found an additional role for this gene in body size determination. Epistasis analysis places AKIR-1 in the TGF-β Sma/Mab pathway; *akir-1**(**gk528**)* mutants display an increase in autophagy markers, a property in other mutants with small body size due to TGF-β signaling perturbations. AKIR-1 impacts developmental processes through the NuRD complex, which is consistent with its role in regulating the innate immune system in *C. elegans* (Polanowska *et al.* 2018).

### Evolutionary conservation of Akirin's function

Akirin is a nuclear protein evolutionarily conserved from worm to fly to mouse [(Goto *et al.* 2007; Komiya *et al.* 2008; Polanowska *et al.* 2018) and this study]. The functional conservation of Akirin’s role in muscle differentiation from fly to mouse prompted us to investigate whether *C. elegans* AKIR-1 was also involved in muscle development and function. Our data indicate that AKIR-1 is important for proper development of muscle tissue in the *C. elegans* embryo and for muscle function in the adult where it is also important for maintaining the integrity of the muscle tissue in aging worms. The movement defects found in *akir-1* mutants are likely due to defects in muscle development, but we cannot rule out an additional role in the nervous system that contributes to this phenotype. As evidence to this conserved role in muscle development, *akir-1* is also required for the proper expression or localization of HLH-8, a transcription factor specifying the muscle M-linage in *C. elegans*. While depletion of *akir-1* results in a reduction of HLH-8-expressing cells, *sma-9* mutation, which also affects M-linage fate, lead to an increase in HLH-8 positive cells instead (Foehr *et al.* 2006). This is in agreement with AKIR-1 and SMA-9 regulating different subsets of genes in the TGF-β Sma/Mab pathway. HLH-8 is the ortholog of Twist (Wang *et al.* 2006). Interestingly, fly Akirin also interacts with Twist in muscle development, suggesting that this interaction is evolutionarily conserved (Nowak *et al.* 2012). Unlike other organisms, there is so far no evidence that AKIR-1 acts in the SWI/SNF-PBAF/PBAP complex pathway in *C. elegans*. Our results instead support a role for AKIR-1 in the NuRD chromatin remodeling complex [as in (Polanowska *et al.* 2018) and this study]. Supporting our hypothesis that AKIR-1 and NuRD work together, mutants for members of the NuRD complex confer a similar set of phenotypes as observed in *akir-1* mutants, including defects in movement, reduced body size and defects in vulva development [(Solari and Ahringer 2000) and this study].

In mouse, the two Akirin orthologs have functionally diverged such that Akirin2 is required for embryonic viability while Akirin1 has non-essential functions (Goto *et al.* 2007). Deletions in the single copy of Akirin in fly leads to late stage embryonic lethality (Nowak *et al.* 2012). However, deletion of *akir-1* in *C. elegans* results only in mild (∼15%) levels of embryonic lethality [this study and (Clemons *et al.* 2013)]. Although the surviving embryos display developmental defects, as observed by the mis-localization of markers for muscle development, many of them proceed successfully through embryogenesis. The *akir-1* mutant embryos that hatch show phenotypes consistent with muscle impairment (movement and egg laying defects), but remain viable with the exception of animals in which the vulva eventually bursts. The degree in which Akirin function is required for proper muscle development and function is likely divergent between worms, flies, and vertebrate models.

### Akirin and the TGF-β Sma/Mab signaling pathway

Studies using fly and mouse systems identified Akirin as a downstream effector of the NFkB/Relish pathway (Goto *et al.* 2007). In this pathway, Akirin may act as a bridge between NFkB/Relish and the BRG1/BRM SWI/SNF chromatin remodeling complex. BRM also interacts with Akirin and Twist during muscle development in the fly, which suggests that a similar transcriptional program may act in response to both muscle development (unknown upstream effectors) and immune system signaling (NFkB/Relish). However, Akirin also interacts with 14-3-3β to negatively impact gene expression in rodents, as opposed to the largely positive regulation imposed by the BRG1/BRM complex (Komiya *et al.* 2008). These studies suggest that Akirin may not be an obligatory partner of SWI/SNF remodeling complexes but could act in a different pathway(s) and mechanism(s). This hypothesis is consistent with our findings and those arising from studies of the immune system in *C. elegans* (Polanowska *et al.* 2018).

We have shown that *akir-1* epistatically interacts with components of the TGF-β Sma/Mab signaling pathway in *C. elegans*. The TGF-β Sma/Mab signaling pathway involves a large number of signaling inputs (receptors and their ligands) and outputs (transcription factors and target genes). Although we have no direct evidence of the specific receptor-ligand interaction acting upstream of AKIR-1, it is likely that DBL-1 is a ligand and type I receptors are involved. This could be inferred from the ability of DBL-1 overexpression to partially suppress body length defects of *akir-1* mutants. This role in promoting transcription is conserved in Akirin proteins in other organisms and consistent with our findings, which places AKIR-1 as one of the downstream components of the TGF-β Sma/Mab signaling pathway. Akirin functions in transcription through interacting with chromatin remodelers. Our data and that of others place Akirin as an interactor with the NuRD complex, suggesting that its role in TGF-β signaling may be through its interactions with the NuRD chromatin remodeler. TGF-β signaling was shown to involve SWI/SNF-dependent chromatin remodeling (Gaarenstroom and Hill 2014). Our genetic analysis, however, suggests that in *C. elegans* NuRD replaces SWI/SNF as the main complex interacting with Akirin suggesting that in worms TGF-β signaling may also involve NuRD.

### Akirin in the germline and in the soma

So far, the role of Akirin in the germline was only reported in *C. elegans* (Clemons *et al.* 2013; Bowman *et al.* 2019). Although the exact molecular mechanism in which Akirin is involved in meiosis is still elusive, the germ cell nuclei in which Akirin mutants’ phenotype is observed are transcriptionally repressed (Walker *et al.* 2007; Merritt *et al.* 2008). This contradicts previously described somatic roles for Akirin in transcription, as a member of chromatin remodeling complexes. However, since Akirin’s function may not be limited to its interaction with specific transcriptions factors, it is also possible that it acts in the germline in ways other than transcription. In our recent study we have shown that AKIR-1 collaborates with IMA-2 nuclear importer to deposit sister-chromatin cohesion in the germline (Bowman *et al.* 2019). Chromatin remodeling is required for formation of a nucleosome-free region that is essential for cohesin loading (Hakimi *et al.* 2002; Muñoz *et al.* 2019). Therefore, we speculate that an Akirin-NuRD nucleosome removal activity could be essential for transcription in the soma, while the same activity will be important for cohesion deposition in the germline.

Our data provide evidence that supports a role for AKIR-1 in embryonic development, likely via somatic expression in muscle and possibly other tissues. This somatic function of *C. elegans* AKIR-1 is consistent with the role described for Akirin proteins in other organisms, acting via the regulation of transcription. Together, these new findings expand the understanding of Akirin function in mitotic and meiotic cells. Further studies investigating Akirin’s functions in the germline of other species will be required to understand whether these roles involve a shared or different molecular pathway.
